# Resin embedded multicycle imaging (REMI): a tool to evaluate protein domains

**DOI:** 10.1038/srep30284

**Published:** 2016-08-08

**Authors:** B. L. Busse, L. Bezrukov, P. S. Blank, J. Zimmerberg

**Affiliations:** 1 Eunice Kennedy Shriver National Institute of Child Health and Human Development, National Institutes of Health, Bethesda, MD, USA

## Abstract

Protein complexes associated with cellular processes comprise a significant fraction of all biology, but our understanding of their heterogeneous organization remains inadequate, particularly for physiological densities of multiple protein species. Towards resolving this limitation, we here present a new technique based on resin-embedded multicycle imaging (REMI) of proteins *in-situ.* By stabilizing protein structure and antigenicity in acrylic resins, affinity labels were repeatedly applied, imaged, removed, and replaced. In principle, an arbitrarily large number of proteins of interest may be imaged on the same specimen with subsequent digital overlay. A series of novel preparative methods were developed to address the problem of imaging multiple protein species in areas of the plasma membrane or volumes of cytoplasm of individual cells. For multiplexed examination of antibody staining we used straightforward computational techniques to align sequential images, and super-resolution microscopy was used to further define membrane protein colocalization. We give one example of a fibroblast membrane with eight multiplexed proteins. A simple statistical analysis of this limited membrane proteomic dataset is sufficient to demonstrate the analytical power contributed by additional imaged proteins when studying membrane protein domains.

Recent advances in microscopy^1^ allow us to localize within cells both individual proteins and some of the interactions between proteins predicted by immunoprecipitation and genetic complementation, but the sheer number of protein species involved in any one biological structure is increasingly becoming a limiting factor. Many-color immuno-histochemistry, while flexible and selective, requires primary antibodies that are either directly coupled to fluorophores or from many diverse species, often forcing a shift to suboptimally affine primary antibodies. Although available immuno-histochemical labels can be supplemented with expressed markers and other methods, their potential permutations still present an overwhelming hurdle for current microscopy methods. Alternatives, such as the expression of tags for subsequent labeling, positively impact the rate at which different molecules can be imaged, but protein colocalization remains speculative with these techniques^2^,^3^. Additionally, techniques exist to enable antibody reuse in unembedded tissue with a variety of elution methods, but the precision of subsequent labeling and tissue damage has not been documented^4^,^5^,^6^.

One promising new technique which introduced procedures for high-dimensional proteomic imaging is array tomography^7^ (ATomo). In ATomo, epitope antigenicity is maintained by embedding a piece of brain tissue into a durable hydrophilic plastic resin which is then cured and subsequently sectioned. Once bound to and protected by such a medium, many protein structures (including desirable epitopes) resist treatments designed to detach or denature antibodies. After the resin cures, newly added proteins (such as antibodies) do not become affixed to the resin but rather bind to the exposed epitopes of the resin-bound proteins of the original sample, via protein-protein interactions that can subsequently respond to environmental shifts in milieu. For example, raising pH to 13 will increase the off-rate of bound antibodies, allowing diffusion into a wash solution while leaving the original epitopes of the sample unchanged and available for relabeling, even at the ultrastructure level^8^. Without resin protection, such high pH would cause irreversible epitope damage. This cycle of label application and removal can be repeated a number of times on the same sample, with different primary antibodies each time.

Cycling of antibody labeling has the effect of greatly reducing the combinatorial complexity of experimentation. For example, rather than generating a new model expressing a tagged variant of a molecule of interest in order to add an extra imaging channel, one can simply remove the current antibodies and apply a new set to label the additional molecule directly. We refer to this physical process as REMI: Resin Embedded Multicycle Imaging.

Here we describe development of new, inexpensive REMI techniques that combine the resilience of resin embedding with the presentation of whole cells and their plasma membranes after isolation as a glass-attached sheet of plasma membrane^9^,^10^. REMI enables studies of membrane protein complexes using multiplexed antibody labeling, effectively creating a single “section” directly from the cells of interest which can then be imaged with the same iterative immuno-staining used in conventional array tomography, in a manner cartooned in Fig. 1. This technique is likely to be useful in studies on the organization of integral membrane proteins in and on the plasma membrane. The advantage of using multiple protein identifications (greater than 2) in a machine learning approach was first described in^11^. Here we demonstrate that multiple protein identifications can be used in an occupancy probability framework to invalidate independent distribution models at lower signal thresholds more robustly. As the number of different protein classes increases, the ability to discriminate between both occupancy and spatial models dramatically increases.

## Materials and Methods

### Preparation of cell membranes

For establishing our technique we used HAB2 cells, a subclone of stably transfected NIH-3T3 (mouse embryonic) fibroblasts that express the hemagglutinin of the A/Japan/305/57 strain influenza virus^12^,^13^,^14^. There is interest in the spatial distribution of HA and other proteins as model systems for the study of membrane domains, membrane fusion, and the assembly of viral particles^15^,^16^,^17^,^18^,^19^. Cells were maintained in Dulbecco’s modified Eagle medium (DMEM, Gibco by Life Technologies, Grand Island, NY, USA) supplemented with 10% fetal bovine serum (FBS, Gemini Bio-Products, Woodland, CA, USA), 100 U/ml penicillin and 10 g/ml streptomycin (Sigma Chemical, St. Louis, MO, USA) in 5% CO_2_. To evaluate REMI using intact cells, both HAB2 and human adipose cells were embedded without membrane isolation.

#### Adhesion

Monolayer HAB2 cell cultures were prepared on poly-L-lysine coated 35 mm dishes with glass bottoms. The shearing procedure for membrane isolation requires cell adherence to the glass substrate; this is achieved through electrostatic attachment of the cells to the positively charged surface of the coated glass^9^. As presented in^10^, to maximize attachment and minimize immunostaining background the dishes were prepared using a 0.25-mg/ml concentration of poly-L-lysine in 0.15 M Borate buffer (pH 8.3) and incubated at room temperature for 30 minutes. To remove excess poly-L-lysine and minimize its toxicity, dishes were washed 5 times with distilled water and once with serum free medium. Cells previously grown to 85–90% confluence were lifted, counted, and seeded in the dishes at concentration of 2.5e5cells/ml in serum free medium. The optimal incubation time for HAB2 cell attachment to the poly-L-lysine-coated glass surface was 2 hours at 37 °C and 5% CO_2_. Under these conditions cells were strongly attached with maximal cell surface adhesion area. After incubating the cellular monolayers for 2 hours, we washed them twice with Intracellular-Like Buffer (ILB) (157 mM L-Glutamic Acid, 13 mM MgSO_4_, 10 mM Pipes, pH 7.3). Adipose cells were also prepared on poly-L-lysine coated dishes following the same protocol.

#### Membrane isolation with shear

To isolate plasma membrane via mechanical cell disruption, a 20 ml syringe with 19 × 1–1/2(TW)A needle was used to generate a stream of ILB that physically sheared the majority of the cells. Dishes were oriented at 60 degrees to the stream and slightly rotated during the process. To remove intracellular debris, we washed the dishes twice with ILB, then verified the cellular disruption by optical microscopy using 20x magnification, repeating the disruption and washing procedure if intact cells remained. To verify plasma membrane integrity the plasma membrane preparation was stained using the hydrophobic fluorophore PKH26 (Sigma Chemicals, St. Louis, MO, USA).

#### Fixation

Attached intact cells or isolated plasma membranes were immediately fixed with freshly prepared 4% paraformaldehyde (PFA) in ILB buffer for 5 minutes at room temperature. PFA was then washed with ILB for 3 cycles of 3 min per wash. To block residual free aldehydes, 50 mM Glycine in ILB was applied for 10 min at room temperature (RT), then removed with 3 wash cycles of ILB at 3 min per wash. When noted, 0.1% glutaraldehyde was added to the fixation mix.

#### Embedding

LR White (LRW) embedding proceeded in a similar fashion to array tomography^20^. Fixed samples were first dehydrated in an ethyl alcohol (200 proof) series: 35%, 50%, 70%, and 90%, (1 × 5 mins each) followed by 100% EtOH (3 × 5 mins) at RT. A solution of 50% LRW/50% EtOH (1 × 10 min) and 100% LRW (2 × 10 min) was used to infiltrate at RT. To obtain a thin layer of LRW prior to polymerization, as much LRW as possible was removed by pipette, then the sample dish was tilted by 45 degrees and more LRW was removed by pipette and absorbent paper (a piece of KimWipe [Kimberly-Clark Sales Inc, Roswell, GA, USA]). Since LRW polymerizes in the absence of oxygen, oxygen was excluded from the ambient air around the LRW by covering the sample with the oxygen barrier film,ACLAR (#50425-10, Electron Microscopy Services, Hatfield, PA, USA). Polymerization occurred in an oven at 60 °C for 24–48 hr.

#### Immunostaining

After polymerization, the samples were washed with ILB. To block non-specific binding, we applied 5%NGS/0.05%TX-100/ILB for 20 min at RT. Upon removing the blocking solution, primary antibody was added in 1/2 blocking buffer for a minimum of 1 hour at RT. Excess primary antibody was removed with ILB washes (3× for 3 min) and secondary antibody was added in 1/2 blocking buffer for a minimum of 30 min at RT, covering the dishes with aluminum foil to minimize light-induced fluorophor degradation. For labeling the plasma membrane, incubations of 30 and 15 minutes of the primary and secondary antibodies, respectively, were generally sufficient. Following the removal of excess secondary antibody with ILB washes (3 × 3 min), SlowFade Gold anti-fade reagent with DAPI was applied to label nuclei and reduce photobleaching, after which the dishes were ready for microscopy.

#### Microscopy

Epifluorescence imaging was conducted on either a Zeiss Axiovert 200 inverted microscope, equipped with a Zeiss 100x/1.45 NA oil-immersion objective, EMCCD camera (Andor iXon EM+, Andor Technology, Belfast, Northern Ireland), and EXFO X-Cite series 120 arc lamp, with a 405 nm, 488 nm, and 561 nm filters or a Nikon Eclipse Ti inverted microscope equipped with a Nikon 60x/1.40 NA PlanApo oil-immersion objective, EMCCD camera (pco.edge 4.2, PCO AG, Kelheim, Germany), and Lumencor Spectra with a Pinkel filter set (Semrock, Rochester, NY, USA). Both microscopes were controlled using Micromanager^21^. To facilitate repeated labeling, samples were secured into the microscope slide holder with transparent tape (Scotch, 3 M, St. Paul, MN). STED imaging was conducted on Leica SP8 STED 3X system (Leica Microsystems), with a white light laser, 592 nm and 660 nm STED depletion lasers and a 100 1.4 NA oil immersion objective lens (HCX PL APO STED white, Leica Microsystems, Mannheim, Germany). Oregon Green and Alexa 594 secondaries were used to enable STED functionality; no other changes to the protocol were required.

#### Elution

Following imaging, the sample was prepared for the next labelling round by applying a solution of 0.2 M NaOH and 0.02% SDS in ddH2O (2× for 10 min), then the elution solution was removed by washing with PBS (2× for 10 min).

### Epifluorescence Image Processing

**Figure 2:**
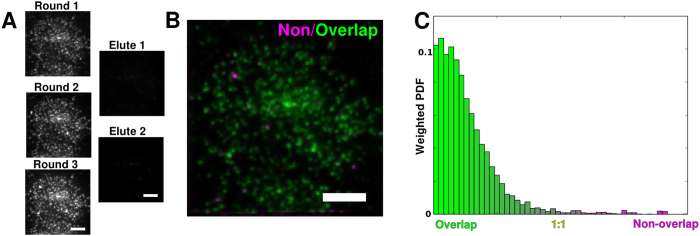
LR-White embedding provides a durable platform for multicycle labeling. (**A**) Immunolabeling of an HA-expressing mouse fibroblast membrane demonstrates epitope preservation and proximity to the surface of the resin. Antibody elution with a solution of 0.2 M NaOH/0.02% SDS/ddH20 (pH 13) for 20 minutes removes fluorescence between rounds. The epitopes survive repeated immunolabeling, their fluorescence increasing slightly after the first elution through an as-yet uncharacterized process of antigen retrieval. This has been observed across different labeled proteins and secondary antibody choices. (**B**) When the average brightnesses are equalized, round 1 and round 3 display a highly correlated labeling pattern (Pearson’s r = 0.89), as illustrated by minmax analysis. (**C**) A color histogram of the previous image, displaying the ratio of green to red, shows that while the two rounds are largely similar, small differences do exist in the relative labeling of puncta. Scale bar: 5 μm.

Image postprocessing was largely conducted using the Fiji image processing software^22^. Fiji’s rolling ball filter was used for background subtraction with the default kernel size of 50 pixels, to eliminate low-frequency background for the multi-round comparisons in Fig. 2.

### Chemicals and solutions

#### Chemicals and solutions used in this experiment comprised

Poly-L-Lysine solution; cell membrane dye (PKH 26 Red Fluorescent Cell Linker Kit); Pipes; L-Glutamic acid; Magnesium sulfate; and TX-100 were all from Sigma Chemicals, St. Louis, MO, USA. Ultrapure water (KD Medical, Columbia, MD, USA); 0.25% trypsin-ethylenediaminetetraacetic acid (EDTA) and NGS-normal goat serum; (Gibco/Invitrogen, Grand Island NY, USA); Glycine (ICN Biomedicals Inc., Aurora, Ohio, USA); Paraformaldehyde and LR White Embedding Resin (Electron Microscopy Sciences, Hatfield, PA, USA); Glass bottom culture dishes (MatTek corporation, Ashland, MA, USA); Boric acid (MG Scientific Inc., Pleasant Prairie, WI, USA); 20% SDS (Quality Biological, Inc., Gaithersburg, MD, USA); Ethyl Alcohol, 200 Proof (The Warner-Graham company (Cockeysville, MD, USA); Aclar (#50425-10, Electron Microscopy Services, Hatfield, PA, USA); Needles (Sherwood medical, ST. Louis, MO, USA); Syringes (Tyco Healthcare Group LP, Mansfield, MA, USA); SealPlate (Excel Scientific, Victorville, CA, USA); Kim Wipes (Kimberly-Clark Sales Inc, Roswell, GA, USA); SlowFade Gold antifade reagent with DAPI (Life Technologies Corporation, Eugene, OR, USA) were used.

#### Primary antibodies used in this work

anti Beta-Actin, Sigma, A1978, AC-15 clone, MoAb, 1:100; anti Beta-Actin, Sigma, A5316, AC-74 clone, 1:100; anti HA, V180, gift from M. Roth, PoAb, 1:300; anti GLUT-4, Abcam, ab654, PoAb, 1:50; Alpha-Tubulin, Invitrogen, A11126, MoAb, 1:50; Anti (Alpha + Beta) Spectrin, PoAb, 1:50, Abcam, ab11182; Anti Alpha Actinin, MoAb, 1:50, Abcam, ab11008; Anti Ezrin, PoAb, 1:100, Cell Signaling, 31455; Anti Alpha Fodrin, MoAb, 1:50, Abcam, ab11750; Anti N-Cadherin, PoAb, 1:50, Abcam, ab76057; Anti GLUT1, PoAb, 1:100, Millipore, 07–1401; Anti B-Actin, MoAb, 1:100, Sigma, A1978, Clone 15; Anti B-Actin, MoAb, 1:100, Sigma, A5316, Clone 74; Anti HA, PoAb, 1:300, gift from M. Roth.

#### Secondary antibodies used in this work

Alexa Fluor 488 goat anti-mouse IgG(H + L), 1:150, A11029; Alexa Fluor 488 goat anti-rabbit IgG(H + L), 1:150, A11034; Alexa Fluor 594 goat anti-mouse IgG(H + L), 1:150, A11005; Alexa Fluor 594 goat anti-rabbit IgG(H + L), 1:150,. A11037; Alexa Fluor 647 donkey anti-mouse IgG(H + L), 1:150, A31571 ; Alexa Fluor 547 donkey anti-rabbit IgG(H + L), 1:150, A31573 (all previous from Invitrogen); Oregon Green, 488 goat anti-mouse IgG(H + L), 06380 (Life Technologies). Nuclei were stained with SlowFade Gold antifade reagent with DAPI, from Molecular Probes, S36939.

### Ethical statement

All procedures were carried out in accordance with NIH experiment guidelines and regulations. Human adipose cells were isolated from tissue samples obtained for the study “Pilot Study of the Effects of Colchicine in Non-Diabetic Adults with Metabolic Syndrome” registered at clinicalTrials.gov (NCT02153983) and approved by the institutional review board of Eunice Kennedy Shriver National Institute of Child Health and Human Development. Human cells were discarded after imaging. No patient information was collected or used in the production of this work.

## Results

### Chamber selection

Rectangular coverslips and surface fluid perfusion are sufficient for labeling ultrathin slices of embedded tissue (e.g. for array tomography), but the embedding of cells in monolayer culture requires a three-dimensional containment device for the various harsh solvents involved in the embedding process. The plastic and glue used in certain commercial glass-bottomed dishes was resilient enough to allow resin embedding of our samples. The use of a dish also proved convenient for handling solutions, as opposed to a constructed well made by hand using a hydrophobic slide marker, as is done in conventional ATomo. In addition, the disposable nature of the dishes meant that they could be discarded once the sample was no longer useful, without having to remove adhered LR White from the chamber for reuse. Capillary action was used to remove excess unpolymerized resin prior to curing.

### Development of anoxic chamber

The principal difficulty in embedding *in-situ* samples in planar form is the need to exclude oxygen. Even small amounts of oxygen prevent LRW polymerization^23^; the gelatin capsules used in array tomography form solid blocks of polymerized resin, but enough oxygen seeps through the gelatin walls to leave a thin surface of unpolymerized resin that is discarded upon block extraction. As our epitopes of interest exist only within a comparably thin surface, all of the resin needs to be polymerized.

We addressed this polymerization problem by filling our chambers with a nonreactive gas: argon. Although argon is heavier than air, we observed mixing with ambient air as soon as the flow of argon gas was stopped, causing poor polymerization marked by tiny bubbles embedded in the resin along with the sample. To solve this mixing problem, we covered the chamber with adhesive film, and used two vents to perfuse the chamber with argon. A small positive flow was maintained until the chamber was sealed completely prior to polymerization. This proved anoxic enough to allow complete polymerization of the LRW. after which the chamber could be returned to ambient air. When successful, the resulting thin layer of resin had a smokey, “rough” texture caused by small perturbations in LRW thickness.

### Functional validation of elution and relabeling capabilities

Though our method produced a thin film of embedded material, the samples’ suitability for proteomic multiplexing needed to be evaluated. Specifically, the thickness of the resin layer required evaluation of small molecule porosity for efficient labeling with fluorescent probes conjugated to IgG secondary antibodies. Smaller molecular weight labels such as PKH26 are assumed to diffuse more easily into the hydrophilic resin, and were not tested. Nevertheless, no barrier to the diffusion of low molecular weight solutes was observed. That the resin layer was thin enough to allow inward diffusion of antibodies was apparently sufficient to permit the labeling of embedded epitopes. Further, the samples were amenable to elution: application of a high-pH elution solution to labeled material reliably stripped 94.5 + /−2% of the signal when applied for twenty minutes. Following elution the sample was relabeled, repeating the process multiple times with the same marker, with imaging after each step. (Fig. 2A).

For some epitopes, relabeling with a new antibody round revealed an interesting phenomenon. Rather than decreasing in intensity or spatially rearranging with elute-relabel repetitions, which may indicate tissue damage, or remain constant through labeling repetitions, we observed an increase in the labeled signal during the first few elute-relabel cycles. For example, when applying REMI to isolated fibroblast membranes, there is a noticeable increase in labeling amplitude between labeling rounds 1 and 3 (Fig. 2A). When the rounds are normalized for intensity, their patterns of punctate labeling are visually similar but not identical (Pearson’s r = 0.89). To visually depict these differences, we developed a straightforward method to generate a two-color image, *I*, where one channel is defined by the original rounds’ (*a* and *b*) shared pixel information, and another is defined by the remaining differences, such that:


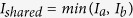



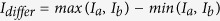


The resulting image is shown in (Fig. 2B). The punctate appearance of the differing (red) material suggests a certain amount of stochasticity in epitope labeling between rounds. We also plot the histogram of similar-difference distributions, (Fig. 2C).

### Multichannel immunolabeling

**Figure 3:**
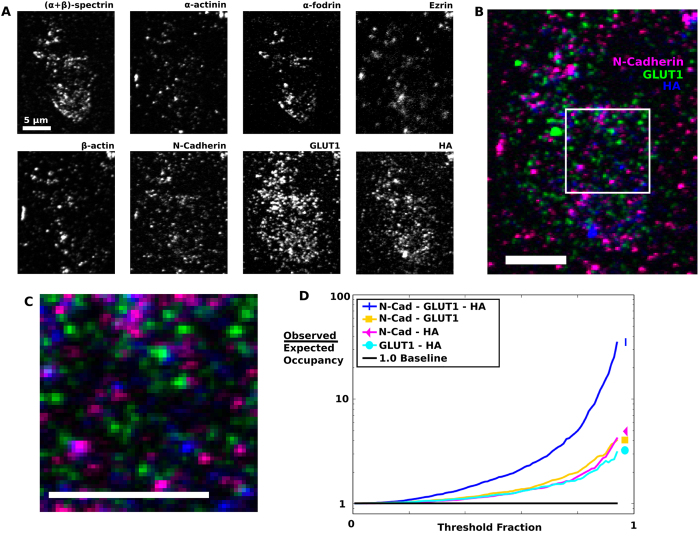
8-channel immunolabeling of membrane and cytoskeletal proteins. (**A**) Heterogeneous immunolabels applied to an isolated HA-expressing mouse fibroblast membrane provide a diverse selection of spatial properties for analysis. (**B**) Three membrane proteins can be compared directly, with an ROI (square inset, (**C**) extracted for numerical analysis. (**D**) When the threshold for analysis is varied across the dynamic range, an exceedance relationship arises, defined as the ratio of pixels occupied by multiple channels to that expected if the channels were independent. Although correlations arise in all comparisons, the triple-occupancy case displays more sensitivity, from lower thresholds, quickly outpacing the doublets. All scale bars are 5 μm.

In order to demonstrate the suitability of REMI embedding to studies involving a multiplicity of proteins, we labeled a single isolated membrane with an assortment of membrane and cytoskeletal protein labels (Fig. 3A). Three of the labels represented proteins not typically associated with the cytoskeleton: N-Cadherin, GLUT1 and HA, and as such their interactions could be compared directly (Fig. 3B,C). Using Otsu thresholding (a common thresholding algorithm which automatically chooses its threshold such that the variance of the pixels above and below the threshold are minimized^24^) to determine the pixel-wise occupancy in each channel, the channels were analyzed in a combinatorial fashion using Chi-squared analysis testing the hypothesis of mutual independence; the channels displayed significant departures from this hypothesis indicating protein-protein interactions (Chi^2^ = 592.9, 4 degrees of freedom, p < 10^−7^).

In order to obtain a more fine-grained analysis without sacrificing the analytical potential of occupancy-based methods, we varied the signal threshold of each channel across the dynamic range, such that from 1% to 95% of the image pixels were below the chosen threshold (Fig. 3D). In the low-threshold regime most of the image was above threshold and was consistent with independence; however when considering triple occupancy, an exceedance relationship, marked by a triple-occupancy count above expectation, quickly became apparent. Pairwise occupancy tests displayed similar relationships, but much less robustly.

### Whole cell and adipocyte embedding

In addition to embedding isolated plasma membranes, our technique was also applied to two types of intact cells: mouse HAB2 fibroblasts and isolated human adipose cells. Aside from the use of whole cells and the elimination of the membrane isolation step, the embedding and imaging procedures were unchanged - cells were fixed and embedded *in situ*.

**Figure 4:**
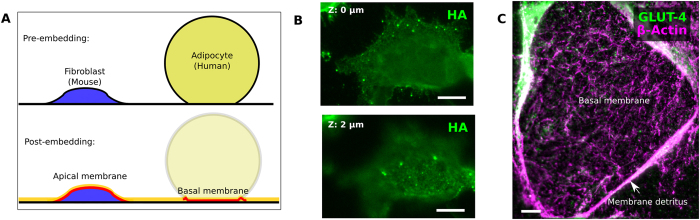
Whole-mount resin embedding of cellular monolayers. REMI also preserves the structure of whole cells, depending on cell type (**A**). “Typical” cells such as mouse fibroblasts (**B**) largely retain their structural integrity, and the LR White resin forms a thin layer above their apical membrane. Human adipocytes (**C**) lose their structural integrity during the embedding process, leaving behind a basal membrane ready for labeling. Scale bar: 10 μm.

HAB2 cells retain their structural integrity after embedding, appearing intact with morphology resembling their pre-embedded state (Fig. 4B). Just as the LRW forms a thin layer encapsulating isolated membranes, here it embeds the cell at least up to its apical membrane, preserving the cell for labeling.

Embedded human adipocytes do not retain their previously spherical shape, but appear to undergo a somewhat dramatic transformation into an isolated plasma membrane surrounded by cellular detritus, exposing the basal membrane for antibody labeling. We infer from their altered appearance that the cells were unable to retain their structural integrity at some point during the course of dehydration and embedding, and deflated. While unexpected, the reproducibility of this result makes for a useful and convenient alternative to membrane isolation followed by embedding, as it shortens the delay between membrane isolation and embedding. This makes the whole-cell embedding preparation, for at least these adipocyte cells, strictly preferable to embedding isolated membranes. It is currently unknown at this time how generalizable this membrane isolation phenomenon will be. It may be entirely limited to adipocytes and similarly sized cells; other cell types might be treated with the aid of physical forces such as osmotic shock to produce comparable membrane isolation.

**Figure 5:**
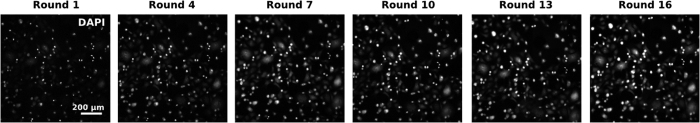
Durability of REMI media. In an extended test of REMI sample durability, mouse fibroblast cells were plated onto a dish with a thin coat of carbon to enhance adhesion, embedded in LRW, and imaged at low resolution with DAPI-containing media (SlowFade Gold). Between each image, the sample underwent three washes of high-pH solution, at 20 minutes per wash, simulating three elution cycles. This was repeated to destruction for each sample; when catastrophic “lifting” of the entire cell layer would occur. Average lifetime of 14 + /−1.6 rounds, n = 3.

For intact embedded cells, the maximum lifetime of REMI was estimated by conducting streamlined relabeling tests of repeated elution. Since epitopes are largely preserved between rounds, evidence of large-scale damage was searched for by labeling with a DAPI-containing mounting medium and using a low power objective. In order to slightly enhance durability, cells were plated onto dishes with a thin layer of carbon, which has been shown to increase binding between LRW and glass^25^. Finally, every third elution round was imaged (one full hour of elution buffer application). These samples survived for an average of 14 rounds, with a 1.6 round 95% confidence window (Fig. 5).

### REMI is compatible with superresolution microscopy

**Figure 6:**
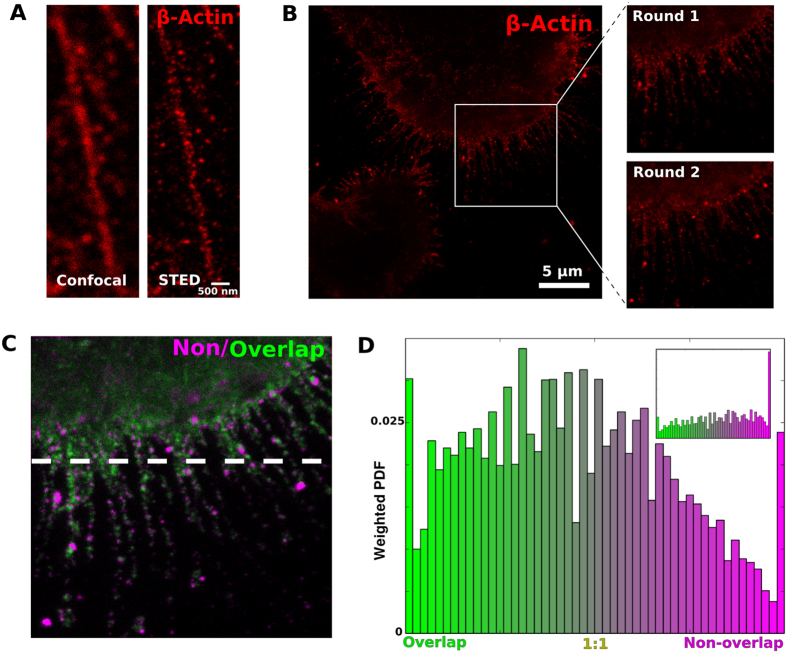
Multiround super-resolution (STED) imaging of embedded cells. The physical properties involved in REMI do not interfere with the functionality of stimulated emission depletion (STED) microscopy of embedded mouse fibroblast cells (**A**). Left: confocal image, right: STED image. Multiple rounds of super-resolved fine structure labeling is also a possibility in STED (**B**), and result in images with similar patterns of epitope labeling, even at such high resolution (inset). Minmaxed analysis of the rounds (**C**) demonstrates a larger degree of epitope fluctuation between rounds, presumably due to stochastic labeling. This becomes evident in the related histogram (**D**). If the region of analysis is restricted to well-defined puncta, as in the bottom half of the image in **C** (below dashes), the stochastic labeling is unmistakable (**D**, inset). Scale bars: 5 μm.

In order to examine labeling reproducibility under REMI in embedded whole-cell conditions, its performance was examined in STED microscopy, which had been previously shown to function with LRW resin^26^. Our primary concern was the impact of STED’s laser fluence, and possible distortion or other deleterious effects on the thin plastic surrounding fine cellular processes. However, in practice no degradation of STED or LRW performance in these conditions was experienced, as evidenced by epitope preservation between labeling rounds (Fig. 6B). On the contrary, in addition to being more visually pleasing, the increased resolution of STED microscopy enabled a number of novel measurements which previously had been unattainable. Principally, STED imaging allowed us to directly visualize the stochastic epitope labeling process, by super-resolving the same epitope sites in successive rounds and submitting the comparison to the same analysis previously used for epifluorescence images. Repeating the stochastic labeling measurement by correlation analysis produced a Pearson’s r of 0.65 in the more finely-resolved context. Repeating the previously described analysis highlighted this effect of increased resolution, more so if the analysis ROI was restricted to just those areas with highly punctate (filopodial) imaging (Fig. 6C,D).

**Figure 7:**
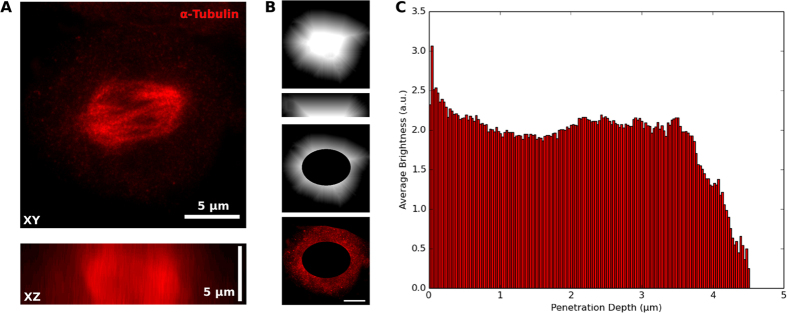
Penetration of immunolabeling in REMI media. A STED volume image of a mouse fibroblast cell, partially rounded while undergoing mitosis, demonstrates antibody penetration when alpha-Tubulin is labeled for precisely two hours. (**A**) averaged projections in the lateral and axial dimensions have visually homogeneous labeling. (**B**) labeling can be mapped onto the distance from the exterior of the cell (lateral and axial profiles, top), optionally removing the bright mitotic spindle labeling (bottom). (**C**) A plot of average intensity as a function of depth within the cell demonstrates uniform labeling inward to the edge of the distance map when the nucleus is excluded. Volume size: 20μm × 20μm × 5.5μm. Scale bar: 5 μm.

The improved spatial resolution of STED microscopy allowed a more accurate estimate of antibody diffusion in LRW, and thus penetration into the material of the cell. Cells which had been labeled with a secondary antibody for exactly two hours and fixed immediately after to prevent further diffusion were imaged at various depths. The most striking examples of penetration were in the nuclei of those cells which had rounded to undergo mitosis, and in which the label had diffused throughout. Labeling was observed at the bottom of these cells, at least 5.5 mm from the nearest surface (Fig. 7). While a proper study of diffusion, stochastic binding, fluorescent activity, and interactions thereof is beyond the scope of this paper, these results demonstrate that IgG secondary antibodies and similarly sized proteins can reach epitopes several microns into the interior of a cell.

## Discussion

### Major findings

Studies of the proteomic organization of the plasma membrane, normally subject to numerous limitations regarding labeling choices, can incorporate resin embedded multicycle imaging (REMI) to obtain information about the complex distributions of membrane protein structures. Epitopes embedded in a hydrophilic resin were protected from high pH washes in that their antigenicity was not significantly diminished between wash rounds. The same pH wash alters the conformation of antibodies bound to those epitopes to sharply increase their off rates and after a short incubation, the original fluorescence is removed (Fig. 2). Epitopes, antibody species, and secondary fluorescence channels are all available for reuse. This technique is compatible with both isolated plasma membranes and intact cells.

In considering the use of REMI for high-dimensional proteomic studies, we can anticipate from array tomography^7^ that panels of eight to twelve proteins, labeled two or three at a time, will provide sufficient channel diversity to enable further studies and novel observations. Since a single round of two to three labels has to-date been the norm for such efforts, analytical methods will need to be improved as well. Our previous work^11^ demonstrates that high-dimensional proteomic data lends itself well to analysis driven by simple feature extraction and classification, although the specific analytical methods may need to be adapted to permit the characterization of diffuse protein regions, or complicated micron-scale membrane complexes. We note, though, that this analysis would be directly applicable to the examination of membrane protein domain formation and structure, which should form nearly punctate regions of interest at the diffraction-limited light level. As such this technique would form a complementary approach to current EM-driven studies of protein clustering in the membrane^27^,^28^ which would stand to benefit from incorporating a diversity of protein species, even at the light level of resolution.

### Label penetration and antigenicity

Although we did not study the dynamics of immunolabeling in detail, the spatial properties of our labeled epitopes allow us to make several inferences regarding the physical structure of REMI-processed samples. Principally, the depth at which we observe labeling implies that label penetration is on the order of microns per hour, and the fact that labeled epitopes are present at all is evidence that the depth to which they are embedded is accessible to diffusion-driven immunolabeling.

An interesting effect we observed in our preparation is the gradual increase in fluorescence over repeated labeling rounds. One explanation, when coupled with the sparse fluorescence remaining after elution, is that the additional signal is merely the leftover label from the previous round, added on to the next. We believe this to be unlikely, as the signal increases too quickly: the intensity of each round is greater than the sum of the previous and its uneluted leftover. The increase more likely indicates some authentic contribution to antigenicity. Given that embedding tissue in LRW imposes an initial decrease in antigenicity^29^, and that our elution protocol contains some elements of the various phenomena known collectively as antigen retrieval^30^ - increased labeling and/or intensity following sample treatment as a result of increased access to epitopes and other binding sites - we suggest that the increase in signal may arise from incidental antigen retrieval during the elution process. This becomes important when considering more quantitative studies that map the raw fluorescence scores onto some other metric. Such studies should apply their antibodies in a consistent order, as well as pretreat the specimen with a round of elution media to invoke the antigen retrieval effect before labeling. Specific testing of each epitope for enhancement may reveal the best strategy for deciding which antibodies to use in which round of labeling.

### Limitations of isolated membranes

An important limitation to the use of membrane isolation prior to embedding involves the delay between isolation and fixation of the plasma membrane. From the moment of isolation, membrane biology is disrupted in an unpredictable manner. For example, decreases in sea urchin cortex cortical granule calcium sensitivity during the first few minutes after shearing have been reported^31^,^32^,^33^.

Immediately upon membrane isolation, some cellular mechanisms continue to function, such as protein diffusion. Others, such as protein insertion and removal from the membrane, do not. As all of these mechanisms are interregulated the disruption of some may influence others during the time between isolation and fixation. The time in which such disruption can take place was limited by applying fixative as quickly as possible, but certain time scales are impractical to outrace effectively. Therefore, we suggest that studies involving isolated membranes will be better suited for experiments in which there is no dynamic deterioration of relevant proteomic structure. If it is important to preserve transient effects or loosely coupled protein relationships, the technique of membrane imaging on whole cells is preferable. Also, if the biology under examination exists in adipocytes or similar cells, whole cell embedding may isolate the basal membrane with less biological disturbance than pre-fixation membrane isolation. Finally, it may be possible to shear the cells with the fixative solution itself using sufficient application force in equipment designed to contain the resulting splatter, as demonstrated in^10^.

### Pitfalls

Although the LRW depth in REMI processing does not appear to interfere with antibody penetration, the physical extent of LRW beyond the cells’ apical surfaces has yet to be determined. It may be possible that varying the LRW thickness will alter the specimen in a useful manner. For example, a thicker LRW coating may positively impact the number of rounds samples can tolerate without incurring epitope damage, while a thinner one may facilitate shorter antibody activation times and quicker label multicycling.

While REMI can provide qualitative assessment of the proteomic composition of protein complexes and other small structures, a more thorough quantitative study would be useful in determining the exact effects of resin embedding on label efficacy and penetration, as well as checking for slow denaturing or detachment processes occurring over multiple imaging rounds. However, such effects are likely to be epitope and label combination specific, so they are best approached with knowledge of the specific protein distribution critical to the phenomenon under examination.

Finally, concerns have been raised regarding the long-term viability of LRW as an embedding medium, due to batch to batch variabilities. While we have encountered no LRW batch problems ourselves, it may be valuable to search for alternative resins, should LRW prove unsuitable for some future work. We have tested REMI methodology using Lowicryl (HM20) (data not shown), and while it demonstrated effective polymerization, its reduced macromolecular penetration^34^ rendered it unsuitable for REMI.

### Potential applications of whole cell surface resin embedding

The successful application of resin embedding to whole cell cultures raises a number of interesting applications. All cell-cell interactions, any binding event, most signal transduction cascades and the majority of neural behavior occurs within a few hundred nanometers of the membrane. These processes are now approachable with REMI. Questions in these important areas can now be approached without the need for labor-intensive construction of expressed labels or biology-disrupting means of membrane permeabilization. Furthermore, the cells are fixed and embedded *in-situ*, without providing any opportunity for cellular biology to be adversely affected prior to fixation. This is in sharp contrast to many preparation methods, which necessarily induce significant changes to cells in order to expose their proteins to imaging.

The performance of embedded HAB2 cells may be interpreted as representative of cultured cells in the generic sense. HAB2 cells are somewhat larger than many other cells types (given sufficient time to attach to the coverslip, they can measure as much as 100 *μ*m from end to end), but the fixation, dehydration and embedding process leaves them apparently intact, with their proteins presumably fixed and preserved *in situ*. This implies that the dorsal portions of many cell types are amenable to analysis with REMI embedding.

### Potential applications to membrane protein domain and raft studies

The organization of proteins in cellular membranes is another research area that attracts considerable interest, yet lacks a framework for the quantification needed to verify current hypotheses. In particular, many features of a general hypothesis termed ‘rafts’ still need to be tested. As outlined by many excellent reviews, the organization of the plasma membrane is conceived as a two-phase membrane with either liquid-ordered domains or liquid-disordered domains, each ranging in size from tens to hundreds of nanometers in diameter, in accordance to the well established demixing of lipids in certain model membranes as a function of lipid composition and temperature. Membrane proteins are then postulated to be mobile in the plane of the membrane and to partition between these two phases depending upon their relative affinity to one phase or the other. Clusters of membrane proteins, according to this raft hypothesis, are due to a high affinity of a protein for rafts.

The multiplexing of protein labels facilitated by REMI methods would be ideal for probing novel hypothetical membrane domain associations. Operating under the assumption that a triple occupancy of proteins is sufficient to define a colocalized protein domain (owing to the low odds of such an occupancy arrangement preserved by chance, as shown in this work), a panel of at least four membrane proteins of interest (we have demonstrated here the ability to image at least eight; although only three were integral membrane proteins) should be sufficient to test those hypotheses which depend on all of the proteins sharing the same raft. Should there be any differences in their mutual association, they should be quantifiable in the form of spatial differences between each triple-occupancy grouping. Six proteins would offer a better comparison, since they could be grouped into two theoretically mututally-independent triplets. More generally, REMI analysis provides a robust platform for exploring the spatial associations of cell surface molecules in other applications, e.g. cellular models for viral assembly^17^ and receptor linkage to the cytoskeleton^35^.

## Additional Information

**How to cite this article**: Busse, B. L. *et al*. Resin embedded multicycle imaging (REMI): a tool to evaluate protein domains. *Sci. Rep.* **6**, 30284; doi: 10.1038/srep30284 (2016).

## Figures and Tables

**Figure 1:**
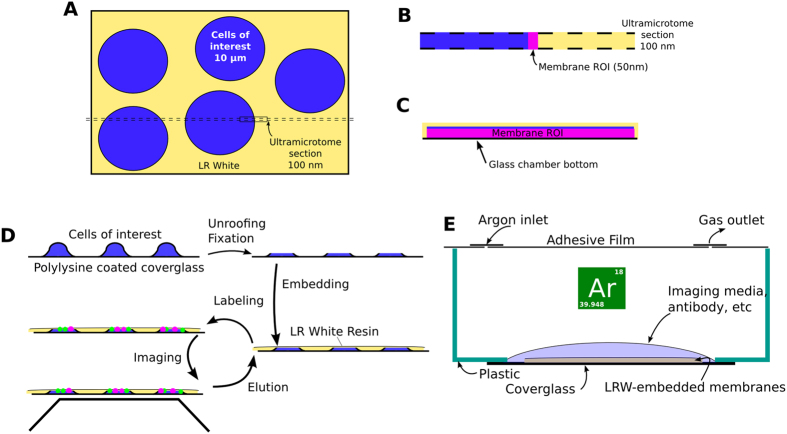
Illustrative depiction of method. A typical array tomography section through cells suspended in LR White (**A**) includes a sizable amount of cell volume, but only fragments of membrane (**B**). We can embed membranes directly to maximize the imageable membrane (**C**). Our method for accomplishing this is cartooned in (**D**). To polymerize in the absence of oxygen, we use a specialized preparation chamber (**E**). A glass-bottomed dish with membrane to be embedded is covered with a sheet of adhesive film. Two holes are punched through the film, used as access points to pump in argon gas and to apply solutions.
